# Network pharmacology to unveil the blood components and mechanisms of Tongmai Yangxin Pills in treating elderly coronary heart disease

**DOI:** 10.3389/fcvm.2025.1475546

**Published:** 2025-02-13

**Authors:** Yue Zhang, Chao-Hui Li, Yi-Zhi Yan, Jie-Yun Lin, Shan-Shan Zhu, Si-Jie Tan, Peng Zeng

**Affiliations:** ^1^ Department of Histology and Embryology, School of Basic Medicine, Hengyang Medical School, University of South China, Hengyang, China; ^2^ Logistics Service Center Medical Office, University of South China, Hengyang, China

**Keywords:** Tongmai Yangxin pills, coronary heart disease, plasma pharmacochemistry, blood components, mechanism, network pharmacology

## Abstract

**Background:**

Tongmai Yangxin Pills (TMYXP) is a well-known traditional Chinese medicine compound to treat coronary heart disease (CHD). Aging is a key immutable independent risk factor for CHD. Currently, there are few gene expression profiles of patients treated with traditional Chinese medicine (TCM) or TCM compound. However, the chemical composition and underlying mechanisms of TMYXP against elderly CHD need to be elucidated.

**Objective:**

Exploring the mechanism of TMYXP in treating elderly CHD based on human gene expression profiles, and find the key pharmacodynamic ingredients of TMYXP in treating elderly CHD based on plasma pharmacochemistry and network pharmacology.

**Methods:**

A strength of this study is the use of network pharmacology analysis of gene expression profiles in elderly CHD patients before and after TMYXP treatment. This study focused on peripheral blood mononuclear cell samples from 6 elderly patients with CHD over 60 years old (GSE142008). A total of 40 blood components of TMYXP identified by UPLC/Q-TOF-MS method in the plasma of SD rats. Then, we collected literature-validated TMYXP blood component targets for further network pharmacology analysis.

**Results:**

All blood components of TMYXP exhibited non-toxic properties. By retrieving validated TMYXP blood components's targets, 15 blood components correspond to a total of 4,789 targets. Genistein, emodin, isoliquiritigenin, glycyrrhizic acid, gallic acid, verbascoside, calycosin, rhein, formononetin and ephedrine were the most potential anti-CHD blood components in TMYXP. The above 10 key blood components of TMYXP mainly regulate hub genes *CASP3, TGFB1, PTGS2, CXCL8, FAS* and *JAK2*, mediating multiple mechanisms to treat elderly CHD. TMYXP exerts anti-CHD effects on the TNF signaling pathway, PI3K-Akt signaling pathway, p53 signaling pathway, MAPK signaling pathway, lipid and atherosclerosis, NOD-like receptor signaling pathway, diabetic cardiomyopathy and cytokine-cytokine receptor interaction. We further used molecular docking technology to verify the direct interaction of TMYXP blood components with its hub target for treating elderly CHD.

**Conclusion:**

This study builds a bridge connecting TMYXP blood components and its confirmed clinical efficacy, identifies a series of anti-CHD lead compounds, and analyzes their possible mechanisms for treating CHD. The research strategy of this study has the potential to promote the modernization and transformation of TCM and promote the drug development.

## 1 Introduction

Coronary heart disease (CHD), a prevalent chronic condition among the elderly, is caused by coronary atherosclerosis narrowing or occluding the lumen, resulting in myocardial ischemia, hypoxia or necrosis (1). Despite significant advancements in disease diagnosis and treatment, CHD continues to be the primary cause of death globally (2). In 2020, the number of CHD patients in mainland China reached 11.39 million, imposing a significant economic burden on society (3, 4). CHD is a multi-factorial disease. Aging is a key immutable independent risk factor for CHD, with its prevalence rising with age in both genders (5, 6). The World Health Organization defines people over 60 years old as the elderly, so this study focused on elderly CHD patients over 60 years old. Given the aging population in China, the burden of cardiovascular disease is expected to rise further. Consequently, it is imperative to identify safe and effective treatments for cardiovascular diseases. Currently, drug therapy is a common treatment method for CHD. Clinically, commonly utilized used CHD treatment drugs include drugs that relieve symptoms and improve ischemia (nitrate drugs, beta-blockers, calcium channel blockers, etc.), and drugs that improve prognosis (anti-platelet drugs, cholesterol-lowering drugs) and traditional Chinese medicine (TCM). TCM has mild effects and few adverse reactions, and patients have good compliance with long-term use (7, 8).

CHD belongs to the categories of “heartache” and “chest arthralgia” in TCM. TCM has a long history and proven efficacy in preventing and treating CHD (9, 10). The TCM prescription Tongmai Yangxin Pills (TMYXP) consists of the classic prescription *Zhigancao Decoction* (炙甘草汤) and *Shengmai Yin* (生脉饮), which is commonly used to treat *Qi* and *Yin* deficiency syndrome caused by CHD and arrhythmia. The expert consensus of the Interventional Cardiology Branch of the China Association of Chinese Medicine recommends the combination of TMYXP and Western medicine for alleviating TCM symptoms, including chest tightness, shortness of breath and palpitations. TMYXP composed of 11 traditional Chinese medicines, including *Radix Rehmanniae*, *Radix Glycyrrhizae*, *Ramulus Cinnamomi*, *Fructus Schisandrae*, *Radix Ophiopogonis*, *Radix Polygoni multiflori preparate*, *Asini Corii colla*, *Caulis Spatholobi*, *Radix Codonopsis*, *Capapax et Plastrum Testudinis*, and *Fructus Jujubae*. In the prescription, Radix Rehmanniae and Radix Glycyrrhizae are the monarch drugs. *Radix Codonopsis*, *Radix Polygoni multiflori preparate*, *Fructus Schisandrae* and *Radix Ophiopogonis* are used as ministerial drugs. TMYXP has been marketed in China for several decades and has proven efficacy in the treatment of CHD (11, 12). At present, TMYXP has entered the local medical insurance catalog in 28 provinces and cities across the country. Clinical studies have shown that TMYXP treatment restored abnormal biochemical indicators in CHD patients and exerted anti-inflammatory effects via the estrogen receptor and NF-κB signaling pathway (11). In the myocardial No-reflow SD rat model, TMYXP reduces myocardial no-reflow by regulating cell apoptosis, and activating both the PI3K/Akt/eNOS pathway (13) and the cAMP/PKA pathway (14). Although the anti-inflammatory properties appear to be the basis of TMYXP's treatment of CHD (11, 15), its blood components and mechanisms for treating CHD remain unclear.

**Figure 1:**
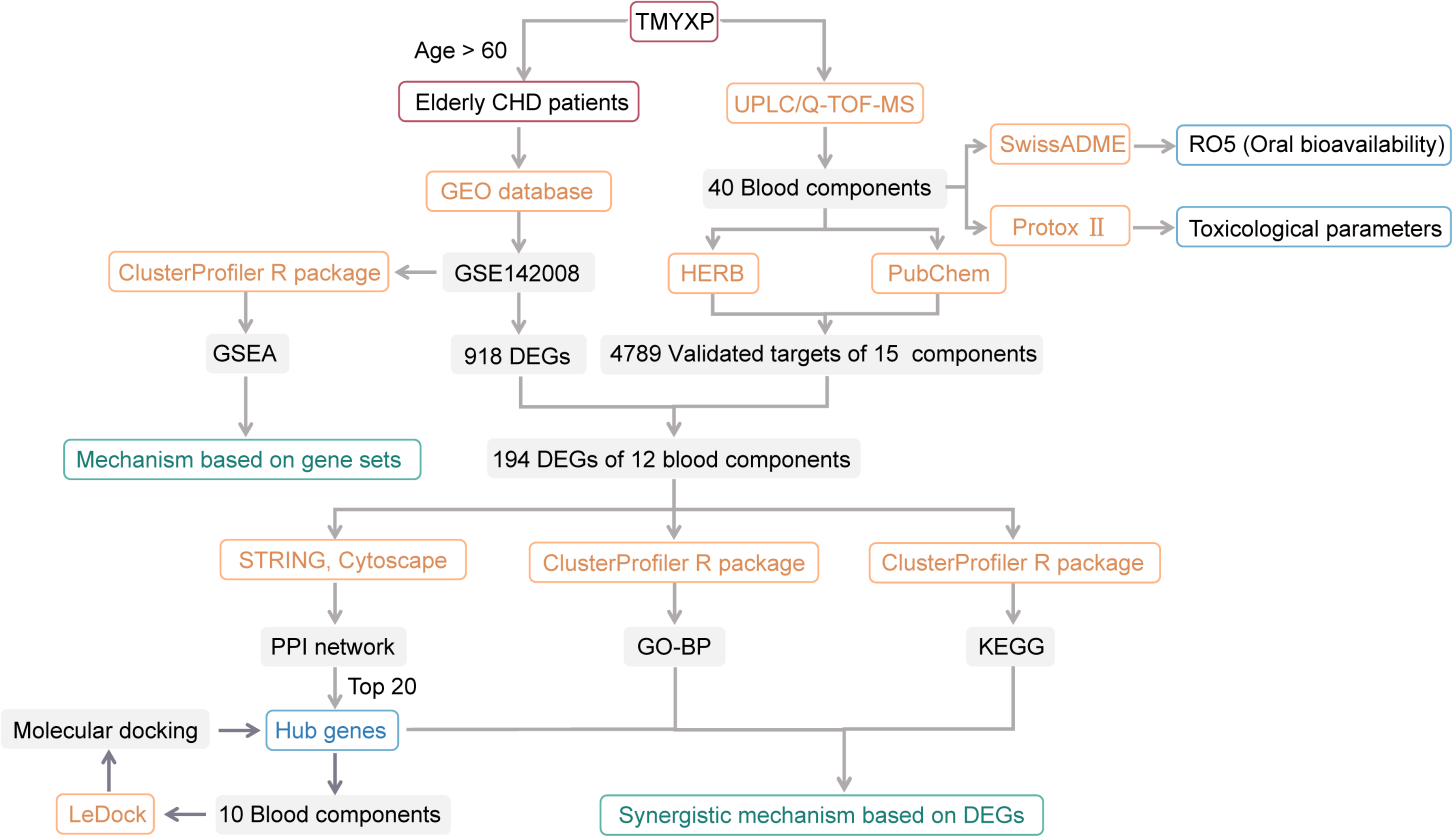
Flowchart of this study. Among the 8 CHD patients in GSE142008, we only focused on 6 elderly patients older than 60 years old. Only 15 of the 40 blood components of TMYXP have literature-validated targets, and only 12 of these 15 blood components have anti-CHD targets.

Network pharmacology is an emerging interdisciplinary field within systematic drug research in the era of big data and artificial intelligence (16, 17). It has created a research paradigm for the transformation of Chinese medicine from empirical medicine to evidence-based medicine, thereby enhancing our understanding of its therapeutic effects (18). TMYXP has a complex chemical composition, containing at least 80 single-molecule components, including flavonoids, coumarins, iridoid glycosides, saponins, and lignans (19). The blood components are the material basis for the efficacy of oral TCM compounds. Therefore, this study focused on the blood components of TMYXP identified by UPLC/Q-TOF-MS method (20, 21). Although these blood components of TMYXP are based on an animal experimental study, their reliability is better than that of components based on database searches of traditional Chinese medicine compound. At present, there are few studies to study the anti-CHD mechanism of TMYXP based on the gene expression profile and plasma pharmacochemistry of elderly CHD patients treated with TMYXP. A notable strength of this study is the application of network pharmacology analysis to gene expression profiles of elderly patients with CHD before and after treatment with TMYXP (GSE142008). The purpose of this study is to build a bridge between the blood components of TMYXP and its efficacy in treating CHD in the elderly, and to clarify the main blood components and mechanism of action of TMYXP against CHD. Figure 1 illustrates the study protocol. This study offers new insights into the mechanism of action of TMYXP in treating CHD in the elderly and provides a series of potential candidate compounds for CHD treatment.

## 2 Materials and methods

### 2.1 Dataset searching and processing

Gene microarray datasets GSE142008 (eight patients with CHD before and after TMYXP treatment for 8 weeks) (15) was obtained from the Gene Expression Omnibus (GEO) database (http://www.ncbi.nlm.nih.gov/geo/); these data were obtained with the GPL570 platform (Affymetrix Human Genome U133 Plus 2.0 Array). This study focused on peripheral blood mononuclear cell samples from 6 elderly patients with CHD over 60 years old (Table 1). This dataset included 16 human peripheral blood mononuclear cells samples of CHD patients, and this study focused on 12 samples from elderly CHD patients older than 60 years old. The limma package in R version 4.2.1 was utilized to identify differentially expressed genes (DEGs) in the before and after TMYXP treatment in elderly CHD patients with cut-offs of adjusted *p* value < 0.05 and |Log_2_(fold change)| > 1. A volcano map was generated using the R package ggplot2 to show the criteria for selecting DEGs. We also conducted principal component analysis (PCA) to cluster peripheral blood mononuclear cell samples before and after 8 weeks of TMYXP treatment.

**Table 1:** Sample information of elderly CHD patients before and after TMYXP treatment.

Sample number	Grouping	Age	Gender
GSM4217594	CHD patients before treatment	61	Male
GSM4217596	CHD patients before treatment	63	Female
GSM4217597	CHD patients before treatment	61	Male
GSM4217598	CHD patients before treatment	66	Female
GSM4217600	CHD patients before treatment	66	Female
GSM4217601	CHD patients before treatment	67	Male
GSM4217602	CHD patients after 8 weeks of TMYXP treatment	61	Male
GSM4217604	CHD patients after 8 weeks of TMYXP treatment	63	Female
GSM4217605	CHD patients after 8 weeks of TMYXP treatment	61	Male
GSM4217606	CHD patients after 8 weeks of TMYXP treatment	66	Female
GSM4217608	CHD patients after 8 weeks of TMYXP treatment	66	Female
GSM4217609	CHD patients after 8 weeks of TMYXP treatment	67	Male

### 2.2 Gene set enrichment analysis (GSEA)

GSEA is a method used to analyze genome-wide expression profile chip data. The analysis was performed using the ClusterProfiler R package (version 4.2.1) (22), which provided the normalized enrichment score (NES) and *p* value for each GSEA.

### 2.3 Collection of blood components of TMYXP and evaluate their pharmacological and toxicological parameters

A total of 40 blood compounds of TMYXP identified by UPLC/Q-TOF-MS method in the plasma (20, 21). The PubChem CID and SMILES of these blood components were downloaded from the PubChem database (https://pubchem.ncbi.nlm.nih.gov/). Compounds meeting Lipinski's rule of five (RO5, MW < 500, Hdon ≤ 5, Hacc ≤ 10, Log*P* ≤ 5 and Rbon ≤ 10) have better oral bioavailability. We used the SwissADME Web server (http://www.swissadme.ch/) to compute the RO5 of TMYXP blood components. ProTox-II webserver (https://tox-new.charite.de/protox_II/) (23) was used to calculate the toxicological parameters of the collected TMYXP blood components, including acute oral toxicity (LD50, mg/kg), hepatotoxicity, carcinogenicity, immunotoxicity, mutagenicity and cytotoxicity. A compound falls into the toxic category if it belongs to an acute toxicity class lower than Class-3 and has more than three active toxicity end points (24).

### 2.4 Collection of validated targets of TMYXP blood components

We used two strategies to collect the validated targets of TMYXP's blood components. Firstly, we downloaded the TMYXP targets that have been reported in the literature from the “Related Gene Targets-Reference Mining” module of the HERB database (http://herb.ac.cn/) (25). Secondly, we downloaded the validated targets of TMYXP from the “Chemical-Target Interactions” module of the PubChem database.

### 2.5 Protein-protein interaction (PPI) network construction and hub gene identification

PPI networks were generated using the STRING database (https://cn.string-db.org/, version 12.0) and visualized with Cytoscape software (version 3.9.1) (26). Interactions with a combined score above 0.4 (indicating medium confidence) were deemed statistically significant in this study. The Network Analysis plugin in Cytoscape was employed to calculate the degree value of each node in the PPI network, where a higher degree indicates greater importance of the protein in the network. The top 20 genes, ordered by degree, were recognized as hub genes in the PPI network.

### 2.6 Gene ontology (GO) and kyoto encyclopedia of genes and genomes (KEGG) pathway enrichment analysis

The enrichment analyses for GO biological processes (BP) and KEGG pathways were performed utilizing the ClusterProfiler R package (version 4.2.1) (22). To account for multiple testing, *p* values were adjusted using the Benjamini–Hochberg method, with statistical significance indicated by a *p* value < 0.05. The enrichment analysis included calculating the rich factor, which represents the ratio of gene numbers to all gene numbers annotated in a specific term. Visualization of the GO terms and KEGG pathways, ranked by the number of enriched genes, was achieved using an online tool (http://www.bioinformatics.com.cn/) (27, 28).

### 2.7 Molecular docking simulation

The molecular docking simulations were conducted using the LeDock program (http://www.lephar.com/software.htm) (29) to assess the interaction between ligands and proteins. The 3D molecular structures of the ligands were downloaded in SDF format from PubChem database and OpenBabel was used to convert the ligand files in SDF format into mol2 format. The structure files of the target proteins in PDB format were obtained from the RCSB Protein Data Bank (PDB database, http://www.rcsb.org/). The LePro tool was utilized to process the receptor files, and the docking score (kcal/mol) was calculated using the default scoring function. The interactions of residues between the receptor and its ligands were visualized using LigPlot (30).

## 3 Results

### 3.1 Analysis of the mechanisms of TMYXP in treating elderly CHD patients

**Figure 2:**
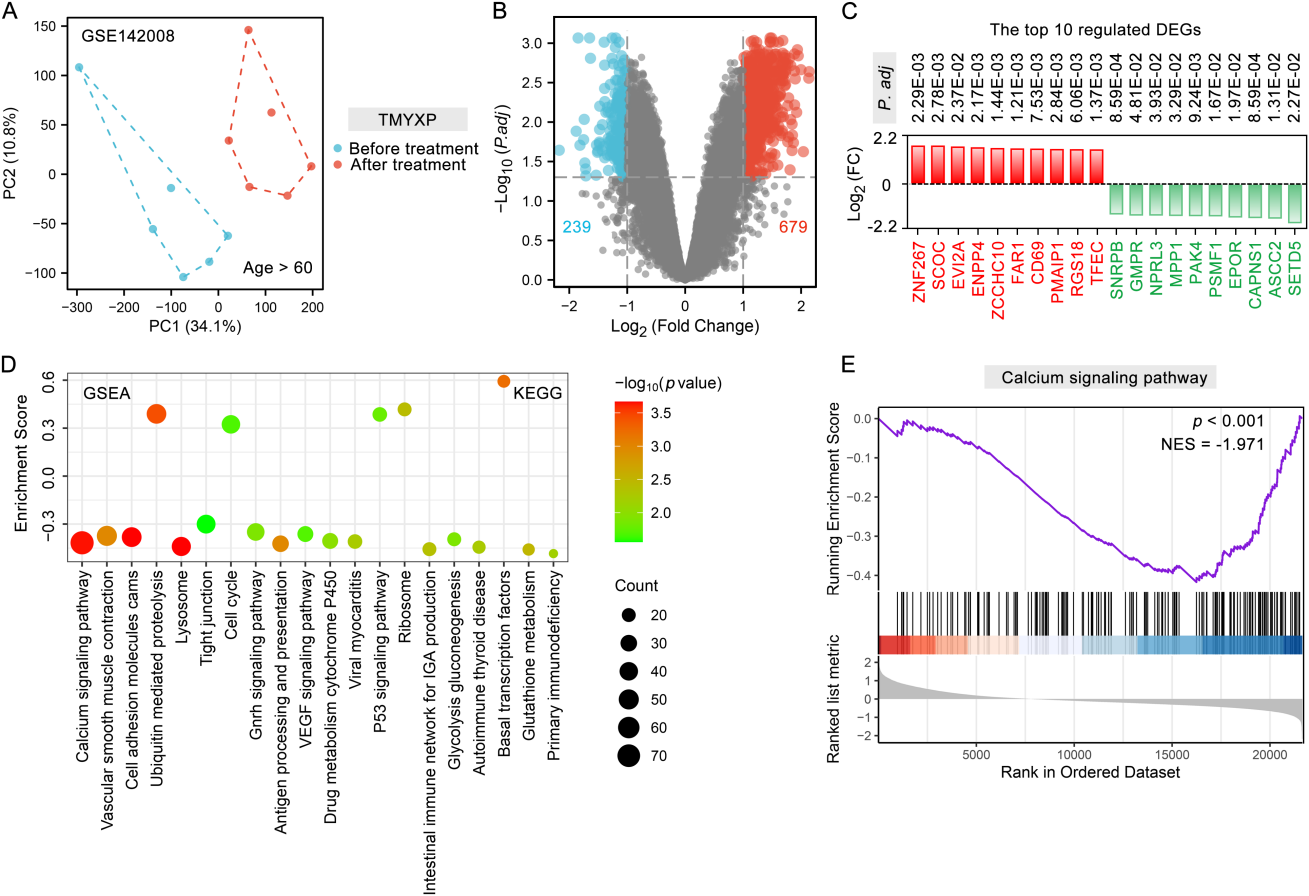
Analysis of DEGs and potential mechanisms before and after TMYXP treatment in elderly CHD patients. **(A)** PCA displays sample clustering within elderly CHD patients before TMYXP treatment and after treatment (*n* = 6). **(B)** Volcano plot showing peripheral blood mononuclear cells DEGs upon TMYXP treatment for 8 weeks. **(C)** Top 10 up-regulated and down-regulated DEGs from CHD patients before and after therapy. Red represents up-regulated, and green represents down-regulated. **(D)** Bubble plot visualization of GSEA for KEGG pathways enriched in elderly CHD patients. **(E)** GSEA plot showing enrichment of calcium signaling pathway signature after TMYXP treatment.

All original data of the network pharmacology analysis is in the Supplementary Materials. PCA analysis demonstrated strong reproducibility within the two groups of samples, along with significant differences between the groups (Figure 2A). A total of 918 DEGs (679 up-regulated and 239 down-regulated) were identified using the criteria of a fold change greater than 2 and a *p*-adjusted value less than 0.05 (Figure 2B). Among these DEGs, *ZNF267, SCOC, EVI2A, ENPP4, ZCCHC10, FAR1, CD69, PMAIP1, RGS18* and *TFEC* were the top 10 most significantly up-regulated DEGs, while *SETD5, ASCC2, CAPNS1, EPOR, PSMF1, PAK4, MPP1, NPRL3, GMPR* and *SNRPB* were the top 10 down-regulated DEGs (Figure 2C). The possible mechanism of TMYXP in treating elderly CHD was discovered using GSEA. A total of 41 signaling pathways were significantly enriched by GSEA (*p* < 0.05). The most anti-CHD TMYXP targets were related to calcium signaling pathway (NES = −1.97, *p* < 0.001), vascular smooth muscle contraction (NES = −1.636, *p* = 0.001), cell adhesion molecules cams (NES = −1.72, *p* < 0.001), ubiquitin mediated proteolysis (NES = 1.70, *p* < 0.001), lysosome (NES = −1.96, *p* < 0.001), tight junction (NES = −1.36, *p* = 0.027), cell cycle (NES = 1.41, *p* = 0.021) and so on (Figure 2D). Calcium signaling pathway is the most significantly enriched pathway, and 74 targets are involved. GSEA plot showing the enrichment of calcium signaling pathway (NES = −1.97, *p* < 0.001) gene set (Figure 2E). The above-mentioned KEGG pathways are important candidate signaling pathways for TMYXP to treat elderly CHD patients.

### 3.2 Blood components of TMYXP and their pharmacological properties

**Figure 3:**
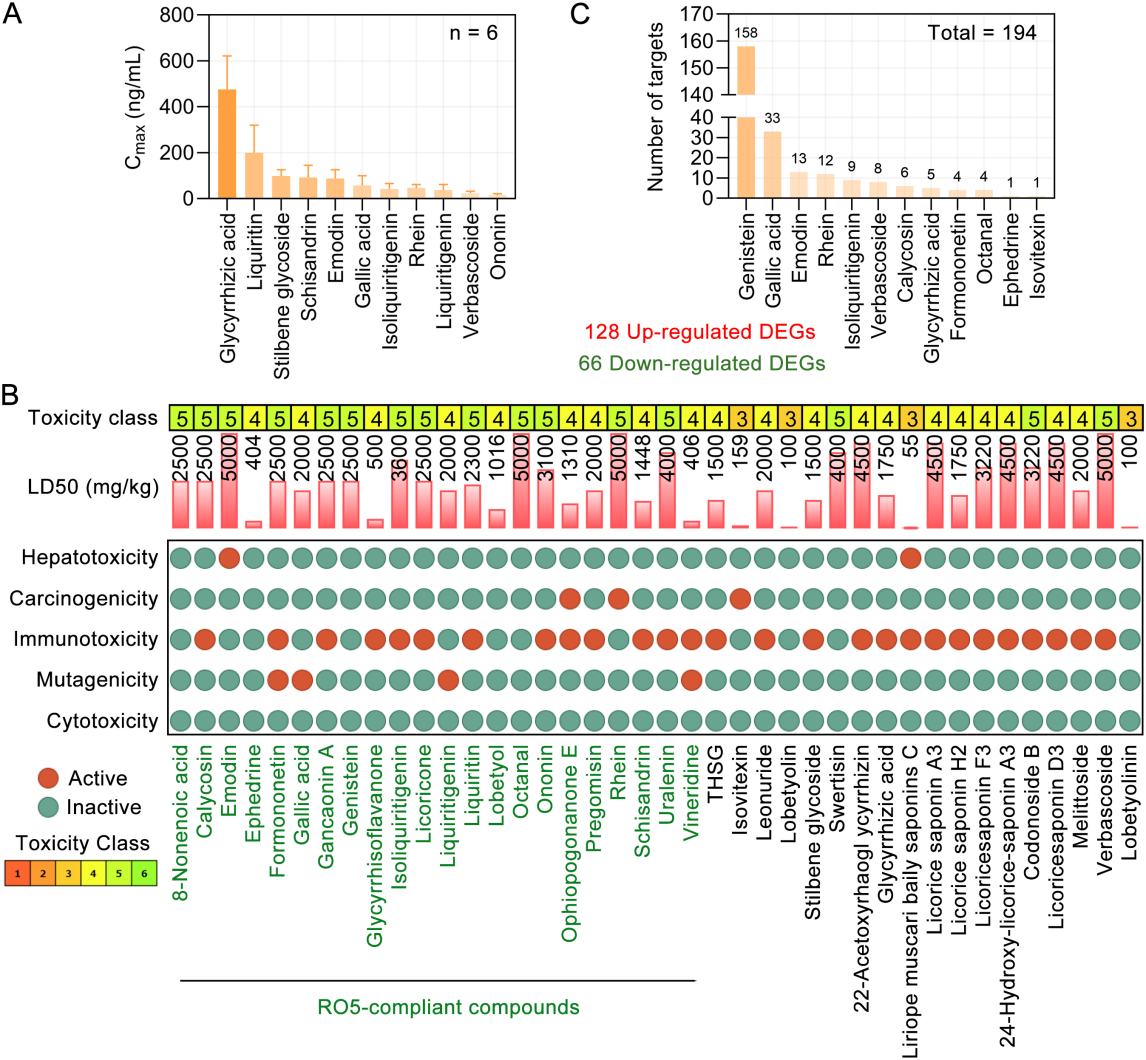
Toxicological parameters of blood components of TMYXP and their DEGs against CHD. **(A)** C_max_ of 11 components in rat plasma after oral administration of a single dose of 8.3 g/kg TMYXP (*n* = 6). Data are represented as mean ± Standard Deviation. **(B)** Toxicological parameters of 40 phytochemicals of TMYXP (20, 21). Phytochemicals of TMYXP shown in green font meet all 5 rules of RO5. **(C)** The 12 blood components of TMYXP correspond to the 194 DEGs of elderly patients with CHD before and after TMYXP treatment.

We focused on the 40 blood components of TMYXP (20, 21), which were identified by UPLC/Q-TOF-MS method (Table 2). After administration of a single oral dose of TMYXP (8.3 g/kg) to normal SD rats, the maximum plasma concentration (C_max_) of the 11 blood components were shown in Figure 3A. Among them, glycyrrhizic acid has the highest C_max_, which is 476.1 ± 146 ng/ml. The physicochemical properties relevant to RO5 were calculated using the SwissADME database (Table 2). Among the 40 blood components of TMYXP, 22 completely comply with RO5 (shown in green font in Figure 3B), and 6 components violate one of the rules. Our findings indicate that these 28 blood components demonstrate favorable oral bioavailability according to RO5.

**Table 2:** Pharmacological and molecular properties of the main blood components of TMYXP.

#	Phytochemicals	Formula	MW/g·mol^−1^	Hdon	Hacc	Rbon	LogP
1	8-Nonenoic acid	C_9_H_16_O_2_	156.22	1	2	7	2.37
2	Calycosin	C_16_H_12_O_5_	284.26	2	5	2	2.30
3	Emodin	C_15_H_10_O_5_	270.24	3	5	0	1.87
4	Ephedrine	C_10_H_15_NO	165.23	2	2	3	1.45
5	Formononetin	C_16_H_12_O_4_	268.26	1	4	2	2.66
6	Gallic acid	C_7_H_6_O_5_	170.12	4	5	1	0.21
7	Gancaonin A	C_21_H_20_O_5_	352.38	2	5	4	3.93
8	Genistein	C_15_H_10_O_5_	270.24	3	5	1	2.04
9	Glycyrrhisoflavanone	C_21_H_20_O_6_	368.38	2	6	2	2.92
10	Isoliquiritigenin	C_15_H_12_O_4_	256.25	3	4	3	2.37
11	Licoricone	C_22_H_22_O_6_	382.41	2	6	5	3.67
12	Liquiritigenin	C_15_H_12_O_4_	256.25	2	4	1	2.07
13	Liquiritin	C_21_H_22_O_9_	418.39	5	9	4	0.25
14	Lobetyol	C_14_H_18_O_3_	234.29	3	3	5	1.74
15	Octanal	C_8_H_16_O	128.21	0	1	6	2.42
16	Ononin	C_22_H_22_O_9_	430.40	4	9	5	0.95
17	Ophiopogonanone E	C_19_H_20_O_7_	360.36	3	7	4	2.35
18	Pregomisin	C_22_H_30_O_6_	390.47	2	6	9	4.07
19	Rhein	C_15_H_8_O_6_	284.22	3	6	1	1.47
20	Schisandrin	C_24_H_32_O_7_	432.51	1	7	6	3.65
21	Uralenin	C_20_H_18_O_6_	354.35	4	6	3	3.13
22	Vineridine	C_22_H_26_N_2_O_5_	398.45	1	6	3	1.78
23	THSG	C_20_H_22_O_9_	406.38	7	9	5	0.39
24	Isovitexin	C_21_H_20_O_10_	432.38	7	10	3	−0.02
25	Leonuride	C_15_H_24_O_9_	348.35	6	9	3	−1.65
26	Lobetyolin	C_20_H_28_O_8_	396.43	6	8	8	0.26
27	Stilbene glycoside	C_20_H_22_O_9_	406.38	7	9	5	0.39
28	Swertisin	C_22_H_22_O_10_	446.40	6	10	4	0.43
29	22-Acetoxyrhaogl ycyrrhizin	C_44_H_64_O_18_	880.97	8	18	9	1.18
30	Glycyrrhizic acid	C_42_H_62_O_16_	822.93	8	16	7	1.49
31	Liriope muscari baily saponins C	C_44_H_70_O_16_	855.02	8	16	7	1.07
32	Licorice saponin A3	C_48_H_72_O_21_	985.07	11	21	10	−0.25
33	Licorice saponin H2	C_42_H_62_O_16_	822.93	8	16	7	1.36
34	Licoricesaponin F3	C_48_H_72_O_19_	953.07	9	19	8	1.06
35	24-Hydroxy-licorice-saponin A3	C_48_H_72_O_22_	1001.07	12	22	11	−0.68
36	Codonoside B	C_58_H_92_O_27_	1221.33	15	27	13	−1.95
37	Licoricesaponin D3	C_50_H_76_O_21_	1013.13	10	21	11	1.11
38	Melittoside	C_21_H_32_O_15_	524.47	10	15	7	−4.07
39	Verbascoside	C_29_H_36_O_15_	624.59	9	15	11	−0.60
40	Lobetyolinin	C_26_H_38_O_13_	558.57	9	13	11	−2.10

THSG, 2,3,5,4′-Tetrahydroxystilbene-2-O-β-D-glucoside.

### 3.3 Evaluation of toxicological parameters of 40 blood components of TMYXP

We utilized the Protox II webserver (23) to analyze the toxicological parameters of these 40 blood components of TMYXP. Our analysis revealed that all blood components of TMYXP exhibited non-toxic characteristics, meeting the criteria of having an acute toxicity class greater than level 3 and less than three active toxicity end points (24). Among these components, emodin, octanal, rhein and verbascoside have the highest LD50 values (5,000 mg/kg), followed by 22-Acetoxyrhaogl ycyrrhizin, 24-Hydroxy-licorice-saponin A3, licorice saponin A3 and licoricesaponin D3 (4,500 mg/kg) (Figure 3B). This suggests that TMYXP has a good clinical safety profile.

By retrieving validated TMYXP blood components's targets, 15 blood components correspond to a total of 4,789 targets. Further intersect the targets of TMYXP with its DEGs before and after treatment in elderly CHD patients, and a total of 194 common targets were identified. Among the 194 DEGs, 128 were up-regulated and 66 were down-regulated. Based on the above 194 anti-CHD targets, genistein has the largest number of anti-CHD targets (158 targets), followed by gallic acid, emodin, rhein and isoliquiritigenin (Figure 3C). These results suggest that TMYXP has the characteristics of multi-components, multi-targets, and multi-pathways.

### 3.4 TMYXP plays a therapeutic role in elderly CHD through multiple mechanisms

**Figure 4:**
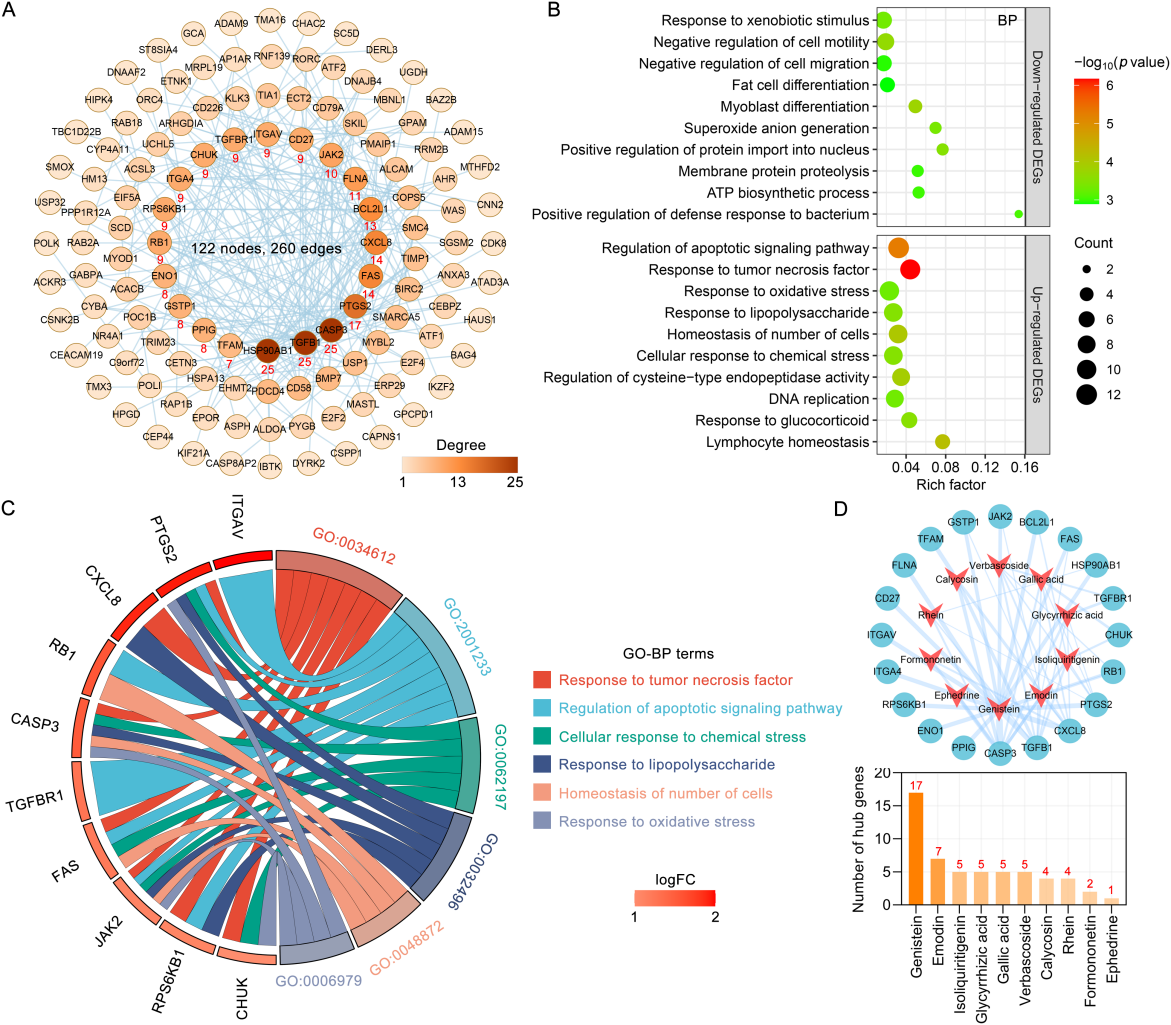
Analysis of hub genes and potential mechanisms of blood components of TMYXP in the treatment of elderly CHD patients. **(A)** The PPI network of 194 anti-CHD targets of TMYXP blood components. Based on their degree values (positively related to node color), the top 20 genes were identified hub genes. **(B)** GO-BP enrichment analysis was conducted to investigate the potential mechanisms of TMYXP blood components in the treatment of elderly CHD patients. *X*-axis, rich factor; bubble size, the number of genes enriched; bubble color, -Log_10_(*p* value). **(C)** Chord diagram of GO-BP involved in 20 hub genes used in TMYXP treatment of CHD. **(D)** The PPI network of hub genes corresponding to the blood components of TMYXP.

The 12 blood components of TMYXP correspond to 194 DEGs before and after TMYXP treatment of elderly CHD. The PPI networks of TMYXP for treating elderly CHD contains 134 nodes and 271 edges (PPI enrichment *p* value < 0.001). The largest PPI network included 122 nodes and 260 edges, as shown in Figure 4A. Based on PPI network, the top 20 highest degree hub nodes, such as *HSP90AB1, TGFB1, CASP3, PTGS2, FAS, CXCL8, BCL2L1, FLNA, JAK2* and *CD27* were selected for hub genes. We then further performed GO-BP enrichment analysis using up-regulated and down-regulated DEGs, respectively. The main enriched BP terms of the 128 up-regulated DEGs were regulation of apoptotic signaling pathway (GO:2001233), response to tumor necrosis factor (GO:0034612), response to oxidative stress (GO:0006979), response to lipopolysaccharide (GO:0032496) and homeostasis of number of cells (GO:0048872) (Figure 4B). The 66 down-regulated DEGs of TMYXP blood components in treating CHD were mainly involved in response to xenobiotic stimulus (GO:0009410), negative regulation of cell motility (GO:2000146), negative regulation of cell migration (GO:0030336), fat cell differentiation (GO:0045444) and myoblast differentiation (GO:0045445) (Figure 4B). Among the hub genes (13 up-regulated and 7 down-regulated), 15 hub genes participated in the 18 GO-BP terms in Figure 4B. There are a total of 6 GO-BP terms containing more than 4 hub genes (Figure 4C). Response to tumor necrosis factor and regulation of apoptotic signaling pathway have the most seven hub genes, and *FAS, JAK2, PTGS2* and *RPS6KB1* were the common hub genes involved in these two BP terms. In Figure 4D, we list the 10 blood components of TMYXP that regulate hub genes. Genistein, emodin, isoliquiritigenin, glycyrrhizic acid, gallic acid and verbascoside were the top six regulators of hub genes.

### 3.5 Molecular docking verification of the hub targets of TMYXP against elderly CHD

**Figure 5:**
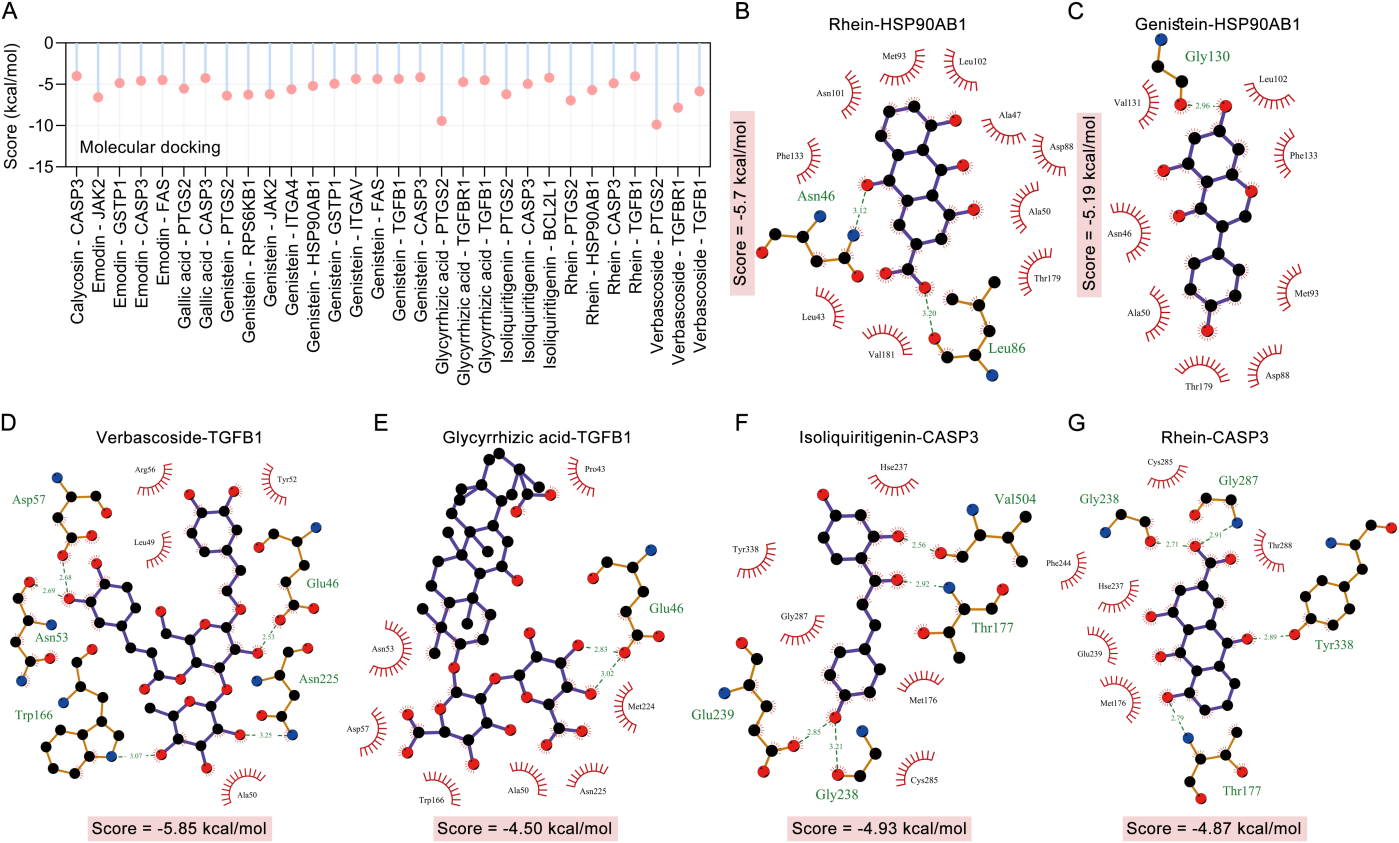
Molecular docking verified the interaction between the hub targets of TMYXP in treating CHD and blood components. **(A)** Bar graph shows ligands and receptors with docking scores below −4.0 kcal/mol. **(B–G)** LigPlus 2D schematic of TMYXP blood component-target interaction. Hydrogen bonds between the compound and the anti-CHD target of TMYXP are indicated by green dashed lines. The structures of HSP90AB1 (PDB: 5UC4), TGFB1 (PDB: 5VQP) and CASP3 (PDB: 1RHK) were obtained from RCSB PDB.

We used molecular docking to verify the direct binding interaction between the blood components of TMYXP and its anti-CHD targets. Among the 10 blood components that TMYXP regulates central genes, 8 blood components correspond to 12 targets with a total of 30 molecular docking scores less than −4 kcal/mol (Figure 5A). Docking results revealed that PTGS2 and verbascoside have the lowest docking scores (Score = −9.87 kcal/mol), followed by PTGS2 and glycyrrhizic acid (Score = −9.42 kcal/mol), TGFBR1 and verbascoside (Score = −7.81 kcal/mol). Subsequently, ligand-protein interactions were analyzed using LigPlot, and only the components with the strongest bindings to HSP90AB1, TGFB1 and CASP3 are depicted (Figures 5B–G). For example, rhein formed potential interactions with residues Asn46, Leu86 of HSP90AB1 through hydrogen bonds, and verbascoside formed potential interactions with residues of Asn53, Asn225, Asp57, Glu46 and Trp166 of TGFB1 through hydrogen bonds. The amino acid residues that form hydrogen bonds with the ligand and the length of the hydrogen bond are shown in green font in the Figure 5.

### 3.6 KEGG pathway enrichment analysis of TMYXP in treating CHD

**Figure 6:**
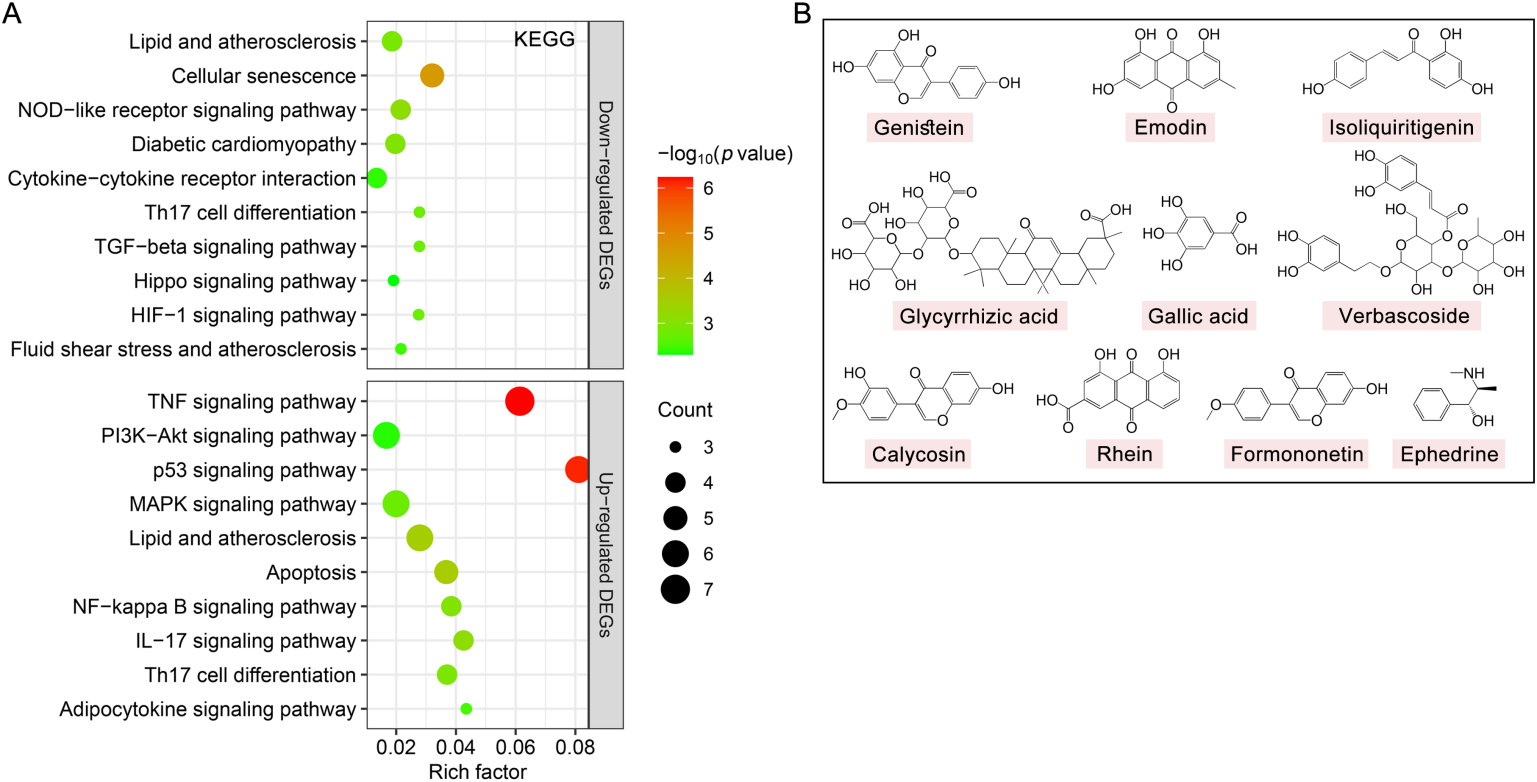
The KEGG pathways affected by TMYXP in the treatment of elderly CHD patients. **(A)** Bubble chart of enrichment analysis results of KEGG pathway analysis. *X*-axis, rich factor; bubble size, the number of genes enriched; bubble color, -Log_10_(*p* value). **(B)** Chemical structures of the key blood component of TMYXP in treating CHD in the elderly.

**Figure 7:**
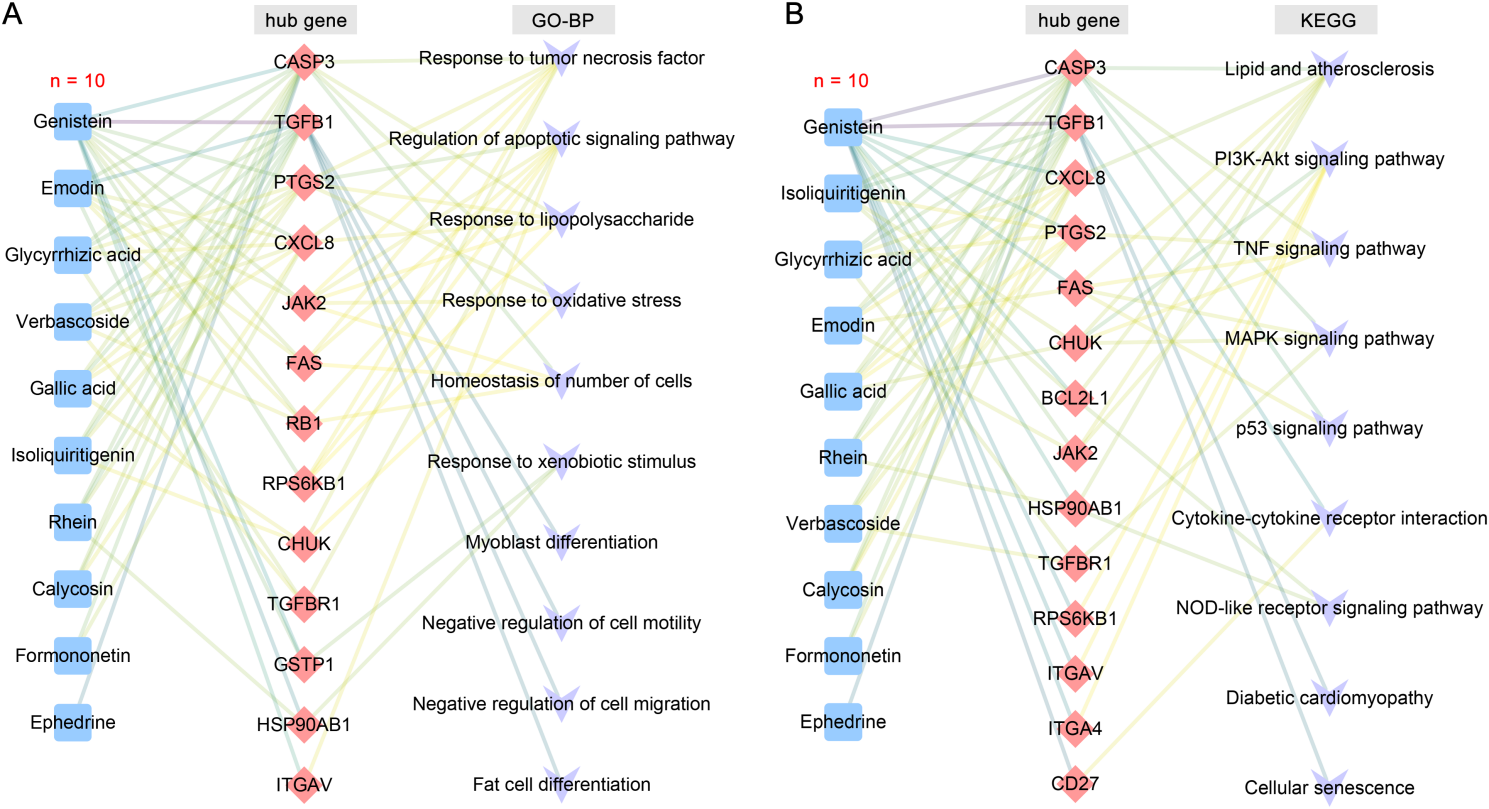
TMYXP's key blood components and mechanisms for treating the elderly CHD. **(A)** The blood components-hub genes-GO-BP network. **(B)** The blood components-hub genes-KEGG pathway network. Blue, red, and purple nodes represent blood components, hub genes, and potential mechanisms, respectively.

We further used KEGG pathway enrichment analysis to reveal the functions of 194 DEGs of TMYXP against CHD. The up-regulated DEGs were mainly enriched in the TNF signaling pathway (hsa04668), PI3K-Akt signaling pathway (hsa04151), p53 signaling pathway (hsa04115) and MAPK signaling pathway (hsa04010) (Figure 6A). Meanwhile, down-regulated DEGs were mainly enriched in lipid and atherosclerosis (hsa05417), cellular senescence (hsa04218), NOD-like receptor signaling pathway (hsa04621), diabetic cardiomyopathy (hsa05415) and cytokine-cytokine receptor interaction (hsa04060) (Figure 6A). The chemical structures of the 10 blood components of TMYXP, which has the most hub genes, were shown in Figure 6B. Based on all the above results, we summarized the hub genes and potential mechanisms regulated by TMYXP's blood components, including the GO-BP and KEGG pathways (Figure 7A,B). The 10 key blood components of TMYXP mainly regulate hub genes *CASP3, TGFB1, PTGS2, CXCL8, FAS* and *JAK2*, mediating multiple mechanisms to treat CHD in the elderly.

## 4 Discussion

In our previous studies (31, 32), we discussed common pitfalls of network pharmacology. This study presents several advantages over traditional network pharmacology approaches. First, we analyzed the underlying mechanism based on the gene expression profile of TMYXP in treating elderly CHD, rather than the disease targets collected in public databases. Currently, there are few gene expression profiles of patients treated with TCM or TCM compound. Therefore, this article uses TMYXP to treat the gene expression profiles of elderly patients with CHD, which significantly improves the reliability of data analysis. Second, the present study primarily concentrated on the 40 blood compounds of TMYXP identified by UPLC/Q-TOF-MS method. The blood components reflect the basis of the efficacy of the oral drugs, which not only increases credibility but also avoids using public databases to screen out a large number of non-specific ingredients. Third, we collected literature-validated targets of TMYXP blood components. Predicting targets based on the chemical structure and drug similarity of compounds can result in both false positives and false negatives. Finally, we also conducted a systematic review of the pharmacological parameters and safety of TMYXP's blood components, which will contribute to the modernization process of TCM.

However, we acknowledge some limitations. First, experimental validation in elderly CHD animal models is needed to confirm our findings. Second, this study focused on the validated TMYXP targets, so the anti-CHD effect of TMYXP blood components without exact targets needs further research. Finally, the gene expression profile this study focuses on involves only 6 elderly CHD patients, and the number of cases in clinical studies needs to be further increased.

Comprehending the intricate relationships among targets, components and pathways is crucial for elucidating the mechanisms underlying TCM compound treat disease. It is also one of the challenges facing the modernization of TCM. Here, key blood components and potential mechanisms of TMYXP in the treatment of CHD were analyzed based on plasma pharmacochemistry and gene expression profile. Genistein, emodin, isoliquiritigenin, glycyrrhizic acid, gallic acid, verbascoside, calycosin, rhein, formononetin and ephedrine were identified as key blood components for TMYXP to treat CHD. Previous research has shown that genistein, formononetin and gallic acid can be used as the main Q-Markers of *Caulis Spatholobi* (33). Emodin is found in both *Caulis Spatholobi* and *Radix Codonopsis*. TMYXP contains 11 compounds with cardiomyocyte protective activity, 2 compounds with angiotensin-converting enzyme (ACE) inhibitory activity, 6 anti-inflammatory compounds, and 4 pro-angiogenic compounds (19, 34, 35). Myocardial ischemia is closely related to vascular endothelial dysfunction. TMYXP reduces vascular endothelial function damage and reduces myocardial ischemic area by regulating apoptosis and activating PI3K/Akt/eNOS and cAMP/PKA pathways (13, 14). The blood components glycyrrhizic acid and licoricone of TMYXP are active substances that protect myocardium. Genistein have been shown to be beneficial in antigen-immunized, allergic, and autoimmune models (36, 37). Previous research has demonstrated that genistein supplementation reduces cardiovascular disease risk factors (38). Furthermore, genistein reduces cardiac mechanical dysfunction caused by glucose toxicity and therefore has the potential to treat diabetes-related cardiac defects (39). The natural active compound emodin is distributed in a variety of medicinal plants and has a variety of biological activities including anti-inflammatory, anti-oxidant, and anti-cancer. Our previous studies showed that emodin improved hyperhomocysteinemia-induced dementia and Alzheimer's disease-like features (40), chronic unpredictable mild stress (CUMS)-induced depressive-like behaviors in rats (41, 42), and neurological deficits in rats with intracerebral hemorrhage (43, 44). Hyperlipidemia is one of the main risk factors for coronary heart disease, and emodin reduces blood lipids through the C/EBP α, PPAR α and AMPK pathways (45). The Huzhang (the root of *Polygonum cuspidatum*)-Shanzha (the fruit of *Crataegus sp.*) is a classic combination of medicinal materials for the treatment of CHD, in which emodin is one of its main medicinal ingredients for the treatment of CHD (46). Previous studies used surface plasmon resonance (SPR), a sensitive method for studying molecular interactions, to detect the interaction between emodin and PPARγ. The results showed that emodin showed high binding affinity to PPAR*γ*, and its equilibrium dissociation constant (KD) was 3.23 μM (47). SPR analysis results also showed that verbascoside and liquiritin showed the best potential binding properties with IL6 and IL1β, respectively (48). The blood components isoliquiritigenin and liquiritigenin of TMYXP significantly enhance DOX-induced H9c2 cell viability, and the mechanism may be related to the PI3K-Akt signaling pathway (49). Future research should further investigate the potential anti-CHD phytochemicals.

The 10 key blood components of TMYXP mainly regulate hub genes *CASP3, TGFB1, PTGS2, CXCL8, FAS* and *JAK2*, mediating various mechanisms for the treatment of CHD. Myocardial cells in a hypoxic state will lead to calcium ion overload, thereby accelerating myocardial cell apoptosis and aggravating myocardial damage. This study identified the calcium signaling pathway as the most significantly enriched pathway in the treatment of CHD with TMYXP. A study showed that TMYXP inhibited calcium ion overload and reduced myocardial cell apoptosis rate.

The effectiveness and safety of TCM compounds are basic requirements for their clinical application. Since its launch 60 years ago, TMYXP has been widely used in clinical practice, with high safety and no adverse reactions detected. After the mice were given TMYXP (equivalent to 240 times the clinical dosage) multiple times within 24 h, no significant toxic reactions were found after continuous observation for 2 weeks. The results of the toxicological parameter analysis in this study indicate that all 40 blood components of TMYXP are non-toxic. Among these components, emodin, octanal, rhein, and verbascoside exhibit the highest safety profiles.

## 5 Conclusion

In summary, our study systematically studied the mechanism and material basis of TMYXP in treating elderly CHD based on gene expression profile, plasma pharmacochemistry and network pharmacology. The research not only establishes a theoretical foundation for the broader clinical use of TMYXP, but also identifies several promising lead compounds for the development of drugs targeting CHD. Specifically, genistein, emodin, isoliquiritigenin, glycyrrhizic acid, gallic acid, verbascoside, calycosin, rhein, formononetin and ephedrine were the most potential anti-CHD blood components in TMYXP. The above 10 key blood components of TMYXP mainly regulate hub genes *CASP3, TGFB1, PTGS2, CXCL8, FAS* and *JAK2*, mediating multiple mechanisms to treat CHD in the elderly. The exploration of classic TCM prescriptions through gene expression profile, plasma pharmacochemistry, and network pharmacology has the potential to advance the modernization and translation of TCM, as well as facilitate new drug development.

## Data Availability

Publicly available datasets were analyzed in this study. This data can be found here: https://www.ncbi.nlm.nih.gov/geo/, accession number: GSE142008.
